# Central African Hunters Exposed to Simian Immunodeficiency Virus

**DOI:** 10.3201/eid1112.050394

**Published:** 2005-12

**Authors:** Marcia L. Kalish, Nathan D. Wolfe, Clement B. Ndongmo, Janet McNicholl, Kenneth E. Robbins, Michael Aidoo, Peter N. Fonjungo, George Alemnji, Clement Zeh, Cyrille F. Djoko, Eitel Mpoudi-Ngole, Donald S. Burke, Thomas M. Folks

**Affiliations:** * Centers for Disease Control and Prevention, Atlanta, Georgia, USA; † Johns Hopkins Bloomberg School of Public Health, Baltimore, Maryland, USA; ‡ Project IRECAM (Investigation of Retroviruses in Cameroon), Yaoundé, Cameroon; § Johns Hopkins Cameroon Program, Yaoundé, Cameroon

**Keywords:** SIV, Bush meat hunters, Cameroon, Central Africa, SIV antibodies, Primate disease, zoonoses, rural populations, risk factors, humans, dispatch

## Abstract

HIV-seronegative Cameroonians with exposure to nonhuman primates were tested for simian immunodeficiency virus (SIV) infection. Seroreactivity was correlated with exposure risk (p<0.001). One person had strong humoral and weak cellular immune reactivity to SIVcol peptides. Humans are exposed to and possibly infected with SIV, which has major public health implications.

Two major public health priorities are ensuring the safety of the blood supply and preventing the emergence of new infectious diseases. Phylogenetic evidence shows that HIV-1 and HIV-2 were introduced into humans through independent cross-species transmission of simian immunodeficiency virus (SIV) strains from distinct, naturally infected, nonhuman primate (NHP) hosts. HIV-1 groups M, N, and O are believed to have arisen as 3 separate cross-species transmissions from chimpanzees, and each of the HIV-2 subtypes A–G was the result of independent transmissions from sooty mangabeys (*Cercocebus atys*) to humans. While laboratory exposure to NHPs has caused infections with SIV (*1**–**3*), no direct evidence has been seen of ongoing exposure to or infection with SIV in natural settings. Nevertheless, hunting and butchering wild NHPs for food, which expose humans to NHP blood and body fluids, are widespread in sub-Saharan Africa and may lead to ongoing transmission from any of the 33 species of NHP that are known to harbor their own unique SIV strains. Since ongoing lentivirus emergence would be of substantial importance to global public health, we looked for evidence of SIV in a unique collection of plasma from persons with known levels of exposure to the blood and body fluids of NHPs (*3*).

## The Study

No commercial serologic assays can detect SIV infections in humans, and published assays for this purpose are not designed to detect a wide range of divergent SIV strains. To determine whether humans are infected with SIV, we developed a sensitive and specific SIV multiple antigenic peptide–based enzyme immunoassay (SMAP-EIA) for detecting *env* IDR (immunodominant region of gp41/gp36) and V3 antibodies to all of the SIV lineages for which *env* sequences were available, specifically SIVsm, SIVagm, SIVsyk, SIVcpz, SIVlhoest/SIVsun, SIVcol, SIVmnd and SIVdrl, SIVrcm, and SIVdeb (*4*). The SMAP-EIA also detects other SIV strains not represented by specific SIV lineage–based peptides.

**Figure 1:**
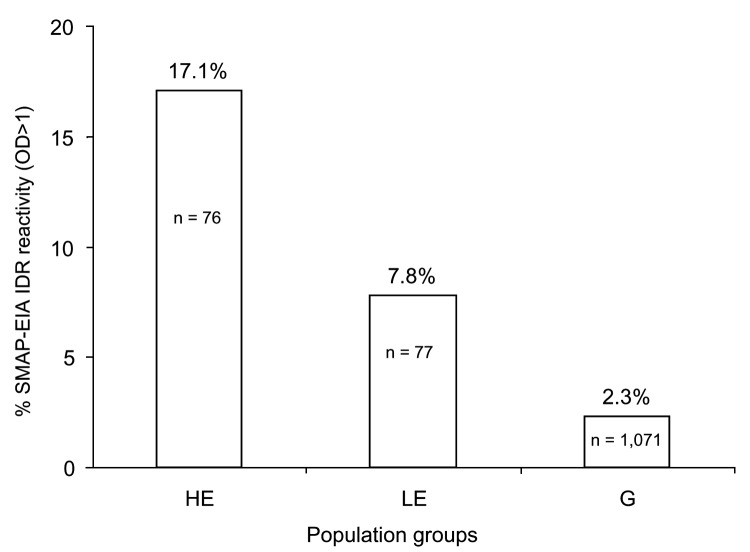
Simian immunodeficiency (SIV) multiple antigenic peptide–enzyme immunoassay (SMAP-EIA) seroreactivity trends to SIV immunodominant region (IDR) peptides in HIV-seronegative Cameroonian population groups with different levels of exposure (high exposure [HE], low exposure [LE], or general [G]) to nonhuman primates. OD, optical density. χ2 linear trend 48.166, p<0.001.

This study was carried out under an approved protocol in accordance with guidelines set forth by the Centers for Disease Control and Prevention (CDC). We tested plasma samples from Cameroon that were seronegative for HIV-1 and HIV-2 by EIA. Cameroon has extensive HIV-1 genetic diversity, and rural bushmeat hunting is common (*2*). Plasma from 3 different groups in Cameroon was examined: 1) persons in remote villages who reported a high level of exposure to SIV strains through hunting NHPs, butchering NHPs, or keeping wild NHP pets (n = 76) (*2*); 2) persons from the same villages who reported a low level of NHP exposure (n = 77) (*2*); and 3) persons from a general population (n = 1,071) from urban and rural areas in Cameroon where people may handle NHP meat but are unlikely to have repeated contact with the blood or body fluids of freshly killed animals. We tested the seroreactivity of these small-volume samples by using our SMAP-EIA. Of the samples that were reactive (optical density [OD] >1.000) to >1 of a panel of 9 SIV IDR MAPs (Figure 1), 17.1% were seroreactive in the high exposure group, 7.8% in the low exposure group, and 2.3% in the general group. The higher the risk for exposure to fresh NHP blood and body fluids, the greater the frequency of reactivity (p<0.001).

**Figure 2:**
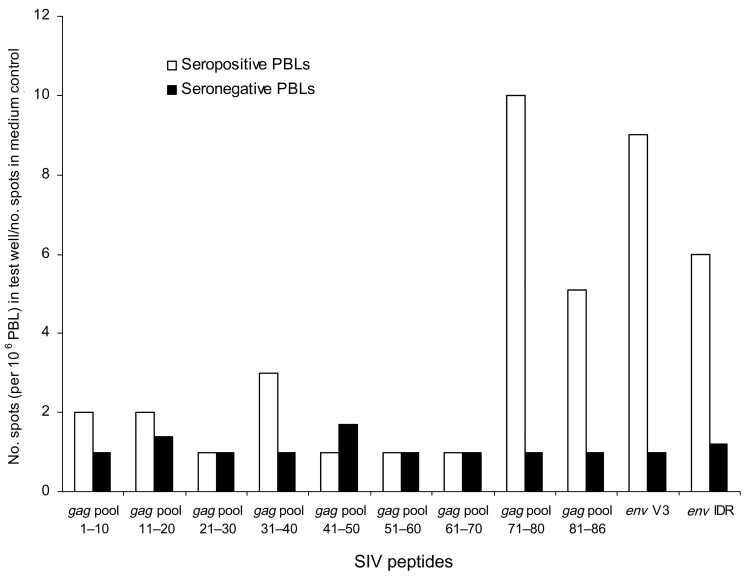
Interferon-γ enzyme-linked immunospot reactivity stimulated with SIVcol peptides from the env and gag regions in peripheral blood lymphocytes (PBLs) from a person seropositive for both the SIVcol V3 and immunodominant region (IDR) peptides and a seronegative person from Africa (both men). To include both assays in a single graph, the number of spots per 106 PBLs for each pool of gag peptides was divided by the number of spots per 106 PBLs in the medium control. This value was expressed as the level of reactivity above background; i.e., the value 2 on the y-axis stands for 2× the number of spots in the negative (medium) control.

Only 1 of the plasma samples, with an IDR OD >1, also reacted strongly to the homologous V3 peptide. This sample, which was from our general population, reacted to the SIVcol (*Colobus guereza*) MAPs in both IDR (OD = 1.250) and V3 (OD = 1.798). Since frozen viable cells were available from this person, we performed an interferon-γ enzyme-linked immunospot (ELISPOT) assay to determine whether peripheral blood lymphocytes (PBLs) from this person recognized SIVcol peptides from *C. guereza*. Since no information is available about T-cell epitopes within the SIVcol genome, and the SIV strains from *C. guereza* are highly divergent from all known SIV isolates (*5*), we designed a series of overlapping peptides (16-mers overlapping by 10) across the *gag* gene, on the basis of the only available *Colobus* sequence (*5*). Pools of 10 peptides were each tested in the ELISPOT assay. Low levels of T-cell reactivity to pools 71–80 and 81–86 of the *gag* peptides (10× and 5× background, respectively, and >25 spots/10^6^ PBLs) and *env* V3 and IDR peptides (9× and 6× background, respectively) were observed with unfractionated PBLs (Figure 2). No reactivity was observed in PBLs from an HIV-1–seronegative African donor used as a negative control. Polymerase chain reaction (PCR) and reverse transcription–PCR amplifications from proviral DNA lysates, plasma from this sample, and cells from stimulated ELISPOT wells were performed with *pol* primers originally used to identify the *C. guereza* sequence (*5*) and with other primers specifically designed from the published *C. guereza* sequence. Despite a strong humoral (*env* IDR and V3) response and weak cellular (*gag*) immune reactivity (in the range of ELISPOT results reported from sex workers who were highly exposed to HIV but seronegative), we were unable to amplify any SIVcol nucleic acids. Seroreactivity without PCR amplification has been documented in those with occupational SIV exposures (*1**,**2*). Therefore, seroreactivity to SIVcol in this person may reflect exposure to nonviable or defective SIVcol, a nonproductive or cleared infection, or sequestering of virus in lymphatic tissues.

## Conclusions

Our data, taken together with previous reports of high prevalence of SIV in NHP bushmeat (*6*) and high levels of NHP exposure (*3*), offer new evidence that persons who hunt and butcher wild NHPs are subject to ongoing exposure and potential infection with SIV. In a study of 16 SIV isolates from 5 different primate lineages, 12 were capable of infecting human monocyte-derived macrophages, and 11 were capable of replicating in human peripheral blood mononuclear cells (*7*), although cell tropism does not necessarily predict virus pathogenicity. Productive crossover infections may occur in low numbers in remote areas of Africa, but because of low population density and isolation, they do not have the opportunity to become epidemic strains and instead become dead-end infections. Ongoing transmission events may also be missed because serologic assays for detecting a broad range of SIVs are lacking or because monitoring is insufficient in populations with high levels of exposure to NHP blood and body fluids. We also have reason to believe that the frequency of SIV exposure and possible infection has increased during recent decades because of a combination of factors that have increased levels of NHP hunting (*3*); these factors include increased access to firearms, increased access to undisturbed NHP habitat from new logging roads, and increased demand for bushmeat in logging camps and rural and urban markets. New roads increase travel, increasing the probability that productive crossover SIV infections will emerge. Further surveillance for new, potentially successful, cross-species lentivirus transmission in Africa is needed to ensure a safe blood supply and prevent the spread of novel, emerging HIV infections.
